# Brain stimulation therapeutics

**DOI:** 10.1016/j.addicn.2023.100080

**Published:** 2023-03-05

**Authors:** Xingbao Li, Mark S George, Abraham Zangen

**Affiliations:** a Brain Stimulation Division, Psychiatry Department, Medical University of South Carolina, Charleston, SC, USA; b Ralph H. Johnson VA Medical Center, Charleston, SC, USA; c Ben-Gurion University of the Negev, Beer-Sheva, Israel

**Keywords:** Smoking cessation, Transcranial magnetic stimulation, Transcranial direct current stimulation

## Abstract

This chapter covers how repetitive transcranial magnetic stimulation (rTMS) or transcranial direct current stimulation (tDCS) presently affects smoking cessation. 14 human studies have examined the efficacy of rTMS on cue craving, cigarette consumption, or smoking cessation using a variety of different coils, locations, and treatment parameters. These studies included 7 randomized-controlled trials (RCT) and 7 experimental studies. Most studies (12/14) reported that rTMS reduced cue-induced craving, 5 showed that it decreased cigarette consumption, and 3/4 reported that multiple sessions of rTMS increased the quit rate. In contrast to rTMS, tDCS has 6 RCT studies, of which only 2 studies reported that tDCS reduced craving, and only 1 reported that it reduced cigarette consumption. Three studies failed to find an effect of tDCS on cravings. No tDCS studies reported changing quitting rates in people who smoke. Despite the early positive results of tDCS on nicotine dependence symptoms, 2 larger RCTs recently failed to find a therapeutic effect of tDCS for smoking cessation. In conclusion, rTMS studies demonstrate that multiple sessions help quit smoking, and it has gained FDA approval for that purpose. However, more studies are needed to examine the effect of tDCS with different treatment parameters.

## 1. Introduction

Tobacco is the most commonly used substance of abuse and one of the main contributing risk factors for diseases [1]. Each year, tobacco causes the death of more than 8 million individuals who smoke. Non-smokers inhaling second-hand smoke constitute about 1.2 million deaths. The high medical expenditures taken to treat tobacco-related illnesses combined with the lost human capital resulting from tobacco-use morbidity and mortality are high economic costs of using tobacco.

In the United States, the number one preventable cause of death is cigarette smoking, accounting for approximately one out of every five deaths [2]. People who smoke pass away 13 to 14 years earlier than people who do not smoke [3]. Tobacco use takes many forms (chewing, smoking, vaping), though cigarette smoking is the most popular, with 45.3 million people (19.3% of the population) who are currently smoking cigarettes. Healthcare costs for smoking-related illnesses are rising, and approximately $96.8 billion is spent yearly on health-related smoking productivity losses [4]. This brings smoking’s total economic weight to nearly $193 billion spent annually.

Most people who smoke do want to quit, but few succeed in stopping. Nearly two-thirds of people who smoke aimed to quit in 2015; over half of them attempted quitting the year prior and sought advice to quit from a healthcare professional. Yet, of these people who smoke attempting to quit, under one-third received cessation treatments and under one in 10 were able to successfully quit within the past year [5]. So, although many different types of therapeutic smoking cessation selections exist, only a small percentage of people who smoke trying to quit (6% out of 35 million) are able to stop successfully for over a month [4,5]. Clearly, there is room for improvement in smoking cessation treatment.

**Fig. 1.**
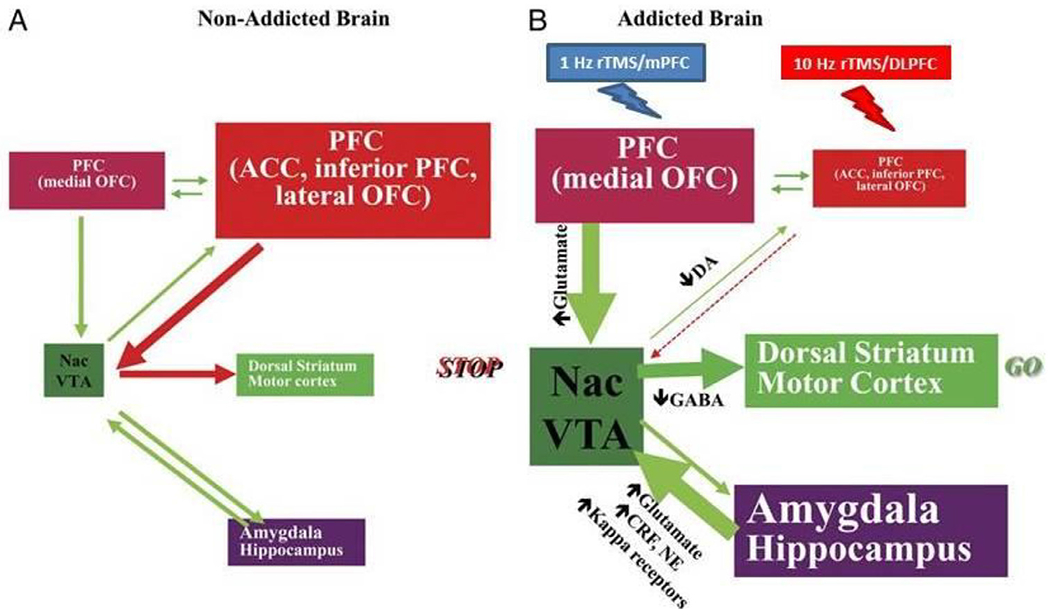
A network of interacting circuits underlying addiction and rTMS-targeted region of interest. ***(A)*** Circuits are balanced in the non-addicted brain. ***(B)*** Drive-reward circuit overcomes the control circuit in the addicted brain. 1 Hz rTMS over mPFC inhibits drive-reward circuits. 10 Hz rTMS over DLPFC increases the control circuit. Modified from Volkow 2011, ref #^6^.

During the addiction process, enhanced drug cues in the memory circuit drive reward expectation and increase motivation to consume the drug, overcoming the inhibitory control exerted by the dysfunctional prefrontal cortex [6] (Fig. 1). When nicotine, smoking tobacco’s psychoactive component, is absorbed, the mesolimbic dopamine system first stimulates the ventral tegmental area (VTA) and then activates reward regions, like the orbital frontal cortex (OFC) and nucleus accumbens (NAc) [7,8]. Fagerstrom Test of Nicotine Dependence (FTND) evaluates nicotine dependent severity, and higher FTND scores are associated with smoking cue-induced brain activation in VTA/substantia nigra (SN), globus pallidus, anterior cingulate cortex (ACC), orbitofrontal cortex (OFC), and temporal cortex [9,10]. A study recently reported that a decreased left dorsolateral prefrontal cortex (DLPFC) blood-oxygen-level-dependent (BOLD) signal could predict smoking relapse [11]. Looking at a different part of the prefrontal cortex, the middle prefrontal, various research projects carried out by our study team at MUSC showed that resisting smoking cravings caused increased brain activity within the bilateral middle prefrontal cortex [12–14]. Therefore, increasing the executive control circuits and resisting the urge to smoke might be developed as a new therapeutical approach for smoking cessation [6,12,15].

Non-invasive brain stimulation (NIBS) has recently surfaced as a novel non-pharmacological treatment for various ailments, including chronic pain, depression, and drug abuse [17]. Brain stimulation treatment’s popularity stems from its ability to change brain function and neural circuitry. This chapter summarizes findings on brain stimulation for treating tobacco use disorder (TUD) and concludes by examining innovative areas for future smoking cessation research.

## 2. Transcranial magnetic stimulation

Transcranial magnetic stimulation (TMS), a type of NIBS, focally stimulates an awake individual’s brain [16,17] by localizing a magnetic pulse to activate the cortex, thereby causing the depolarization of neurons [18] and generating brain electrical currents [19]. Delivering TMS pulses repetitively and rhythmically is called repetitive TMS (rTMS). rTMS has been studied to treat neuropsychiatric diseases (e.g., depression, psychosis, anxiety) [20].

### 2.1. rTMS for smoking cessation

The U.S. Food and Drug Administration (FDA) cleared high-frequency rTMS (HF-rTMS) over the DLPFC for the treatment of depression in 2008 [21,22] and for smoking cessation in 2020 [23,24]. To date, 14 human studies using various treatment parameters have tested the effects of rTMS on cue-induced craving, cigarette consumption, and quitting smoking (Table 1). These studies included 7 randomized-controlled trials (RCT) [24–30] and 7 experimental studies [31–37]. Most studies (12/14) reported that prefrontal rTMS reduced cue craving, 7 studies showed that it decreased cigarette consumption, and 4/5 reported that multiple sessions of rTMS increased the quit rate. Regarding experimental studies, 6/7 are crossover designs.

In one study, investigators gave HF-rTMS over the left DLPFC to treatment-seeking adult smokers [32]. Participants received 20 trains of either sham or real rTMS (20 Hz, 90% motor threshold, 2.5 secs-on, 42.5 s intertrain intervals, 1000 pulses total) over the left prefrontal cortex on 4 consecutive days. While real rTMS was associated with a significant drop in smoked cigarettes per day (CPD) compared to sham stimulation (*p* < .01), levels of craving did not significantly differ across groups.

Rose and colleagues used 10 Hz versus 1-Hz TMS over the superior frontal gyrus (SFG) in 15 volunteers who smoked. They found that the 10-Hz SFG condition increased smoking-cue-induced craving while the control, a neutral-cue-induced craving, did not [36]. These preliminary studies suggested that the location of the stimulation is critical and that HF-rTMS of the DLPFC, but not the medial area, might attenuate nicotine consumption and craving.

In another study, MUSC researchers conducted a controlled experimental study comparing active 10 Hz rTMS vs. sham rTMS over the left DLPFC in two treatment visits, crossover design with a 1-week interval between study visits [35]. Active rTMS over the left DLFPC significantly reduced craving from baseline [35]. Higher FTND scores and more cigarettes smoked each day were positively correlated with greater reductions in subjective craving induced by rTMS [35]. With respect to RCT, 7 RCTs have been done in rTMS for smoking cessation. A parallel group of heavy nicotine-dependent people who smoke with schizophrenia [32] who entered a sham-controlled rTMS trial in combination with wearing the nicotine patch did not show increased abstinence rates. Still, rTMS significantly reduced short-term (30–60 min) abstinence from tobacco cravings, as measured before nicotine patch application.

Amiaz and colleagues reported that HF-rTMS for 10 days on the left DLPFC decreased cigarette intake and reduced nicotine dependence [25]. Another study reported that deep TMS delivered over the DLPFC and insula reduced cigarette consumption and resulted in abstinence after 3-week rTMS treatment [26]. Recently, our randomized, sham-controlled, double-blind clinical trial revealed that 2 weeks of imaging-guided rTMS of the left DLPFC decreased cigarette consumption and enhanced the rate of quitting [27].

The depth of stimulation can affect the clinical effects of rTMS in clinical application. In comparison, the deep-TMS coil induces a magnetic field with larger distribution and depth than does the standard TMS coil, without a significant increase in the intensity of the electric field induced in superficial cortical regions [38]. Recently, the most definitive trial with deep TMS in this field was reported, sponsored by a TMS manufacturer, Brainsway. This multicenter, prospective, randomized, double-blind, sham-controlled clinical trial in outpatient adults who smoke explored the efficacy and safety of the Brainsway Deep TMS (DTMS) coil for quitting smoking. From August 2014 to July 2019, participants signed up in 14 study centers (12 in the United States and 2 in Israel). They randomly received either active DTMS or sham treatment with a 1:1 ratio. For 3 weeks, they received daily TMS sessions at 10 Hz, 120% stimulation intensity of MT measurement, 3-second pulse trains, 15-second inter-train intervals, and 60 trains, registering 1800 pulses each session. The primary efficacy measure was abstinence (0 cigarettes/day), verified by urine cotinine ≤200 ng/ml. 262 participants randomly received either active (123 subjects) or sham (139 subjects) TMS treatment. The 4-week Continuous Quit Rate (CQR) was significantly higher (p-value = 0.0174) in the DTMS group (19.4%) than in the sham group (8.7%). The study’s primary efficacy endpoint was substantiated by secondary endpoints, including 4-week CQR in subjects with at least 4 weeks of diary records, 4-week CQR up to the 6th week visit, and cigarettes smoked per day (diary entry) for all participants. The positive treatment result was demonstrated quickly (as soon as 2 weeks) after treatment commenced. 18 DTMS sessions substantially and significantly increased the quit rate success in people who smoke attempting to quit.

In 2020, the US FDA approved deep rTMS (120% resting MT, 10 Hz in 3-second pulse trains, 15-second inter-train intervals, 60 trains, 1800 pulses total) as a therapeutic treatment for smoking cessation [39]. Recently, at MUSC, we reported that 5 sessions of rTMS decreased tobacco consumption and cue-craving in lung cancer patients who smoke [28]. All things considered, these earlier studies show that HF-rTMS over the left DLPFC can reduce cigarette intake [25,27,32], and craving [27,35,40], and boost the quit rate [25–27].

Recently, a brand-new kind of rTMS known as theta burst stimulation (TBS) was created [41]. TBS is a form of TMS that uses theta frequency and a bursting pattern to stimulate the brain. Theta burst may be more efficient in terms of time and effectiveness since it employs the same frequency that the brain communicates with its own circuits. Unfortunately, no studies have supported that TBS is more efficient than standard high-frequency rTMS for depression treatments [42]. TBS’s main advantage is its rapidity of administration, despite the fact that it was once believed to provide more potent and repeatable results than other rTMS techniques. TBS treatments can be applied in 2–3 min or less, which makes them more convenient for participants than lengthy protocols like traditional 10 Hz rTMS, which can take 37 min. [43]. Intermittent TBS (iTBS), one type of TBS, provides 600 pulses in just three minutes, each at a frequency of 50 Hz and repeated at intervals of 5 Hz, demonstrating a similar response as conventional standard rTMS [44]. According to another study, adding iTBS to psychotherapy enhanced nicotine cessation [45]. Of late, a different study found that iTBS enhanced inhibitory control but continuous TBS (cTBS) decreased it [46]. Additional research is required to determine how TBS affects smoking cessation in tobacco use disorder.

### 2.2. rTMS and brain circuitry

Although the exact mechanism of rTMS for smoking cessation is unknown, it can be theorized that it modifies brain circuits governing neurobiological processes that underlie addiction, such as drug seeking, reactivity, risk-reward, and decision making–or that it inhibits pre-potent responses [47,48]. Magnetic pulses can depolarize superficial neurons, which causes electrical currents in the brain to focally excite the cortex [20,49]. Several studies have demonstrated that stimulating the motor cortex with different frequencies of TMS can produce inhibitory (low-frequency, ≤1 Hz) or excitatory (high-frequency, ≥5 Hz) effects [50,51].

Volkow and colleagues [6,52,53] have proposed a systems model of addiction for substance use. According to earlier research, the drive-reward and executive control circuits must be balanced for proper inhibitory control and decision-making. Recently, our studies demonstrated that the left DLPFC HF-rTMS treatment increases brain activity in the prefrontal cortex and inhibits brain activity in the mesolimbic reward system (mOFC and NAc) in TUD [34,54]. These findings suggest that repeated DLPFC stimulation could enhance executive function and decrease drug seeking by elevating DLPFC and ACC activity and lowering mOFC and NAc activity. Furthermore, the elevated activity in the middle prefrontal cortex correlates with “resisting” the urge to smoke [12–14]. Therefore, by targeting the DLPFC, using 10 Hz rTMS can normalize the hypoactive DLPFC, causing people who smoke to inhibit impulsive smoking behaviors (Fig. 1).

A more active mOFC is associated with higher saliency of rewards or cues and craving [6,52,53]. Several previous research projects carried out by our team have reported that brain activation of the left medial prefrontal cortex relates to smoking cue-induced craving in TUDs [12,14,55]. In another study, participants who were effective in reducing cravings had lower mOFC metabolism [56]. Recently, Hanlon and colleagues reported that 6 sessions of continuous theta-burst stimulation (cTBS) produced a long-term, depression-like effect on the frontal pole, subsequently inducing a marked reduction in orbitofrontal activity [57]. Therefore, by targeting the mOFC with LF-rTMS, 1 Hz rTMS can normalize the hyperactive mOFC, directly causing people who smoke to decrease desiring or wanting cigarettes. (Fig. 1)

A previous study reported that TUD is strongly associated with the insula [58]. Insula activity in cigarette cravings may have something to do with decision-making [59]. The H-coil stimulated the bilateral insula and was FDA-approved as a treatment option for tobacco use disorder [24,26,39,60]. Further neuroimaging study is needed to determine if the H-coil effects necessarily involve the insula in tobacco use disorders.

## 3. Transcranial direct current stimulation

tDCS, another NIBS method, has been tested to treat substance use disorders. Unlike rTMS, tDCS utilizes a weak and direct current (typically 1–2 mA) to flow unilaterally between two electrodes on the scalp. tDCS can be applied un-hemispherically or bi-hemispherically, targeting dual stimulation to brain regions. Cortical excitability has been discovered to be modulated by tDCS in research on animals and people [61]. Some suggest that cathodal stimulation reduces excitability while anodal stimulation increases excitability [61,62]. Through tDCS, spontaneous neuronal firing is directly altered: anodal tDCS initiates cortical depolarization, whereas cathodal tDCS causes hyperpolarization [63,64]. tDCS has unclear mechanisms of action, but it accurately influences neuronal excitability differentially and generates spontaneous neuronal firing, making it an attractive treatment for psychiatric illnesses with known cortical excitability alterations, like depression [65]and tobacco use disorder [66].

### 3.1. tDCS for smoking cessation

Impulsive behaviors are closely linked to drug use and abuse, both as contributors to use and as consequences of use. Trait impulsivity is an important predictor of drug use during development. Increased impulsive behavior may make drug use more likely [67] . Previous studies have shown that tDCS reduces impulsive and risk-taking behaviors in people who smoke [66,68]. However, there have been inconsistent findings related to the efficacy of tDCS for smoking cessation. Furthermore, tDCS is not FDA-approved for an indication, though trials have demonstrated potential efficacy in treating depression and other disorders.

To date, 6 RCT studies [68–73] and 5 crossover-experiment studies [66,74–77] have used tDCS for smoking cessation (Table 2). Within the 6 RCT studies, only 2 reported that tDCS reduced craving, while 1 reported that it reduced cigarette consumption. However, the effects of tDCS on cravings were not detected in the other three studies. No studies reported quitting rate changes in people who smoke after tDCS treatment. There are some negative findings despite the earlier positive results of tDCS on the symptoms of smoking dependence. Recently, 2 RCTs that looked for tDCS’s therapeutic impact on tobacco use disorder were unsuccessful [70,73]. However, these studies were likely under-dosed, as only 6 sessions and 3 sessions were delivered to the participants. Within 5 crossover-design studies, 4 studies reported that tDCS reduced cigarette craving [66,74,76,77] and 2 reported reductions in cigarette consumption [74,77]. The inconsistency and variability noted in previous studies can be due to several factors; varying study designs (e.g., duration, length, intensity, and location target of treatment) are likely primary factors contributing to these discrepancies. For example, previous studies showed that the median number of tDCS treatment sessions for depression was 10, applied over the course of 2 weeks [78]. Notably, when cathodal tDCS activated DLPFC areas, the tDCS procedures had considerable positive effects on cue-induced craving and tobacco consumption. The site of the cathodal stimulation was diverse (e.g., DLPFC and supraorbital area), and even though the anodal tDCS target employed in other investigations, the DLPFC, was largely identical. According to earlier research, desire states might be linked to a balance between the activity of bilateral DLPFC. By using cathodal tDCS over either the left or right DLPFC, any disruption of this equilibrium could decrease the intensity of craving [79]. In summary, greater effectiveness was obtained by anodal stimulation on the left DLPFC and cathodal stimulation on the right DLPFC [80].

In summary, more recent investigations [70,73] have failed to confirm older studies’ [71,74,77] effectiveness. However, both studies had a lower number of therapy sessions, in one study, participants received 3 sessions, in the other study, they received 6 sessions in three days within one week. It is unclear whether more treatment sessions can improve the treatment efficacy of tDCS for smoking cessation. Furthermore, 6 of 11 studies (Table 2) indicated that tDCS reduced tobacco craving, while 3 of 11 studies reported that tDCS decreased cigarette consumption. Recently, a meta-analysis paper demonstrated that anodal tDCS stimulation over the left DLPFC in combination with cathodal stimulation over the right DLPFC provided higher treatment efficacy [80]. Therefore, future research should potentially increase the stimulation session number with stimulating anodal stimulation over the left DLPFC and cathodal stimulation over the right DLPFC.

### 3.2. tDCS and brain circuitry

tDCS is used as a NIBS technology to explore basic neuroscience research and clinical application for various neuropsychiatric diseases since it has the ability to change brain connectivity. According to a recent study, tDCS might have an impact on two significant core networks linked to nicotine withdrawal syndrome [81]. Researchers showed that Anode-tDCS to left DLPFC in a single session amplifies the salience network’s (S.N.) upregulation and the default mode network’s (D.M.N) downregulation at working memory and that these results are amplified in nicotine-satiated people who smoke. Caudate nucleus subcortical dopamine release may be enhanced by stimulation of the DLPFC, increasing the involvement of a crucial brain region in reward and cognitive regulation [82]. Using a particular type of tDCS, like high-definition tDCS, could possibly be a potent tool for changing deeper brain networks, which would have a stronger impact on modulating cue-craving and smoking-intake patterns [83].

## 4. Conclusion and future perspectives

rTMS and tDCS are two of the most used NIBS methods for quitting smoking. Since these methods are non-invasive, painless, and may be used with electroencephalogram (EEG), functional magnetic resonance imaging (fMRI), and other technical tools to evaluate their utility, they are popular amongst researchers and medical professionals. In psychiatric settings, they are both commonly used. However, differences exist between them and should be considered when choosing a preference for research. tDCS is less expensive than rTMS and easier to use [79]. Yet, recent RCT trials of tDCS contradict claims that it reduced smoking and reduced cue-induced desire [70,73]. In comparison, rTMS is more effective (Table 1) and has received FDA approval to be used for quitting smoking. Future research needs to investigate various tDCS protocols that affect and increase the efficacy of tDCS for smoking cessation.

Several theories can explain why brain stimulation is effective in helping people stop smoking. One of the primary cerebral regions thought to be involved in addictive behaviors seems to be the prefrontal cortex; prefrontal inhibitory dysfunction is the main underlying cause of addiction [6,52]. Nucleus accumbens, which are at the center of addiction neurocircuitry, receive dopaminergic neurons from VTA. In substance use disorder patients, neuroimaging studies have revealed decreased activity in certain brain regions between episodes of craving. High-frequency rTMS and tDCS (anode stimulation) applied across the left DLPFC can both enhance cognitive function and modify brain activity in the region [84,85]. In addition, the insula and mOFC are important regions for managing smoking cessation [24,34]. Therefore, focused brain stimulation on certain brain regions may enhance treatment effectiveness for quitting smoking. Future studies should also contemplate the following problems: (i) which brain regions (e.g., DLPFC, insula, or mOFC) and (ii) which hemisphere (left, right, or both) work best, considering (iii) it might be interesting to integrate behavioral therapy and NIBS in prospective management, as well as (iv) investigate whether TBS can result in more effective treatment and (v) develop accelerated rTMS and tDCS protocols while (vi) combining TMS and tDCS techniques together.

## Figures and Tables

**Table 1:** Summary of published TMS studies and preliminary data for Tobacco Use Disorder.

Study name	N	SUB	Study Design	Tx-Seeking	Target method	Target site	Sham method	Sessions \pulses	TMS parameter	Outcomes		
										Craving	Consumption	Quit
Eichamner (2003) [32]	14	HS	Crossover	Yes	5 cm to M1	Left DLPFC	Sham coil	4\4000	20 Hz 90%MT	NS	↓	UR
Amiaz (2009) [25]	48	HS	RCT	Yes	5 cm to M1	Left DLPFC	Mu plate	10\10,000	10 Hz, 100%MT	↓	↓	12.5%
Rose (2011) [36]	15	HS	Crossover	No	Fixed 10/20	SFG	M1 tms	3\1500	1 Hz, 10 Hz 90%MT	↓	UR	UR
Wing (2012) [30]	15	SZP	RCT	Yes	Fixed 10/20	Left /right DLPFC	90 ° coil	20\15,000	20 Hz, 90%MT/NT	↓	UR	UR
Li (2013) [35]	16	HS	Crossover	Yes	6 cm to M1	Left DLPFC	E-sham	2\6000	10 Hz, 100%MT	↓	UR	UR
Hayashi (2013) [33]	10	HS	Crossover	No	MRI guided	Left DLPFC	90° coil	1	1 Hz, 59% output	↓	UR	UR
Prikryl (2014) [29]	35	SZP	RCT	No	5 cm to M1	Left DLPFC	Sham coil	21\42,000	10 Hz, 110%MT	↓	↓	UR
Dinur-Klein (2014) [26]	115	HS	RCT	Yes	6 cm to M1	PFC/insula	Sham coil	13\12,870	1 Hz, 10 Hz 120%MT	↓	↓	33%
Trojak (2015) [37]	37	HS	Crossover	Yes	MRI guided	Right DLPFC	Sham coil	10\3600	1 Hz, 120%MT/NRT	↓	UR	NS
Li (2017) [34]	10	HS	Crossover	No	6 cm to M1	Left DLPFC	E-sham	2\6000	10 Hz, 100%MT	NS	UR	UR
Chang (2018) [31]	10	HS	Open	Yes	MRI guided	Left DLPFC	No	10\10,000	20 Hz, 90%MT	↓	UR	UR
Li (2020) [27]	42	HS	RCT	Yes	MRI guided	Left DLPFC	E-sham	10\30,000	10 Hz, 100%MT	↓	↓	24%
Zangen (2021) [24]	262	HS	RCT	Yes	6 cm to M1	Right and left DLPFC and Insula	Sham coil	18/1,8000	120%MT	↓	↓	19%
Li (2022) [28]	11	CS	RCT	Yes	6 cm to M1	Left DLPFC	Sham coil	5\15,000	10 Hz, 100%MT	↓	↓	UR

*Note:* SUB – subjects, HS- healthy smoker, SZP – schizophrenia patients, CS – cancer-currently-smoking, RCT- randomized controlled trial, Tx –treatment, E-sham – electrical TMS sham, MT – motor threshold, NS – not significantly changed, UR – unreported, NRT – nicotine replacement.

**Table 2:** Summary of published tDCS studies and preliminary data for tobacco use disorder.

Study Name	Sample size	Study Design	tDCS protocols							Outcomes		
			Sessions	A-tDCS	C-tDCS	Intensity (mA)	Duration (min)	Size (cm^2^)	Sham	Craving	Consumption	Quit
Fregni 2008 [66]	24	Crossover	3	R-DLPFC	L-DLPFC	2	20	35	y	↓	UR	UR
Boggio 2009 [68]	27	RCT	5	L-DLPFC	R-DLPFC	1	20	30	y	↓	UR	UR
Xu 2013 [75]	24	Crossover	2	L-DLPFC	R-DLPFC	2	20	35	y	NS	NS	UR
Fectean 2014 [77]	12	Crossover	2	R-DLPFC	L-DLPFC	2	30	35	y	↓	↓	UR
Meng 2014 [71]	30	RCT	3	Bi-OF	Bi-PFT	2	20	Diameter 6.5	y	NS	↓	UR
Falcone 2016 [74]	25	Crossover	2	L-DLPFC	R-SOB	1	19	25	y	↓	↓	UR
Yang 2017 [76]	32	Crossover	2	L-DLPFC	R-DLPFC	2	20	35	y	↓	*UR*	UR
Mondino 2018 [72]	29	RCT	10	R-DLPFC	L-OL	2	20	35	y	↓	NS	UR
Alghamdi 2019 [69]	22	RCT	3	R-DLPFC	L-DLPFC	1.5	20	25	y	UR	NS	UR
Falcone 2019 [70]	106	RCT	3	L-DLPFC	R-SOB	1	20	25	y	NS	NS	NS
Verveer 2020 [73]	73	RCT	6	R-DLPFC	L-DLPFC	2	20	35	y	NS	NS	UR

*Note:* RCT- randomized controlled trial, NS – not significantly changed, UR – unreported.

## Data Availability

No data was used for the research described in the article.

## Abbreviations:

NIBS: Non-invasive brain stimulation
TMS: Transcranial electrical stimulation
tDCS: Transcranial direct current stimulation
